# The Manchester Royal Infirmary

**Published:** 1895-04-06

**Authors:** 


### THE INSTITUTIONAL WORKSHOP.

### HOSPITAL CONSTRUCTION.

### THE MANCHESTER ROYAL INFIRMARY.

The people of Manchester find themselves in a difficulty. They have a hospital which, besides being deficient in many ways, is far too small. To extend it, however, in a satisfactory manner upon the present site is impossible, without sinning against the very canons of hospital construction. The site, moreover, is so valuable that it would be easy by the sale of it to provide a new one on which a perfect hospital could be built. Besides all this, the trustees decided at a meeting held on February 26th that complete rebuilding of the Royal Infirmary was necessary, although at a subsequent meeting held on March 26th it was determined to consider the matter further before a final decision was arrived at. Everything then would seem favourable to a completely new start, and the erection of a sanitary building on a sanitary site. And yet, under the glamour of old tradition, and influenced, perhaps, by a not unnatural dislike to see an old landmark swept away, they are on the verge of doing what will always be to their discredit, and will remain a monument, standing in the very midst of their city, marking the decadence of public spirit in its inhabitants.

Before it is too late, the people of Manchester should bear in mind the splendid opportunity which now lies within their grasp, if they will but put sentiment and prejudice on one side. The present buildings are of no value for hospital purposes, for rebuilding is already practically decided upon; the site, however, is of great value, and this gives the chance of obtaining a new one in the best possible position. An opportunity such as this only occurs very rarely either to an institution or an individual. Unfettered either by buildings or by site, the people of Manchester are absolutely free to act for the best. In building and in placing a hospital for the treatment of the sick then, what is best? The essentials for the erection of a really satisfactory hospital are space, pure air, isolation of ward from ward, and facility of access. Under no circumstances can sufficient space be obtained on the present site, nor is there the slightest hope, judging by the observations made in Manchester during recent years, that there will be any improvement in the character of the atmosphere in the centre of the town. The impossibility of isolation of ward from ward in any building erected on the present site is an even more serious matter. Under stress of circumstances, and because they have been driven to second-rate courses by the extravagant price of land, people in London and elsewhere have erected hospitals in which a considerable number of beds have been crowded upon a small area, and we will quite admit the necessity for such a course where, as in London, many miles would have to be traversed before a more open situation could be reached. It has been recognised all the time, however, that this crowding of beds was bad.

It has been a choice of evils; the lesser one has, no doubt, been accepted, but it has been, and is, admitted on all hands that, with equal care in management and construction, that hospital will be the most health-giving in which but few people are lodged under one roof. Everywhere the most advanced teaching and practice in the construction of hospitals is in favour of breaking them up into many blocks, every ward receiving light and air on both sides, and every block being capable of absolute isolation from its neighbours; and it is a curious thing that it should be left for Manchester to advocate the retrograde step of building a hospital with back-to-back wards, seeing that from Manchester and its medical officers has come the most scathing criticism of back-to-back houses, and that Manchester was one of the very first towns in England to adopt the system of completely-isolated pavilions in hospital construction, as exemplified in the workhouse infirmary at Withington.

The figures given by Messrs. Young and Hall, when they were consulted on the matter in 1891, are worthy of attention. They show that if the required additions were placed upon the present site the area of site per bed would be about 400 square feet, whereas the average site-area per bed of 41 hospitals in London and the largest provincial centres, including Edinburgh and Glasgow, is 877 square feet, while on the Continent the average site area of 21 hospitals amounts to no less than 1,368 square feet. Perhaps the Manchester people, accustomed to their tall warehouses, think that sick folk can be stored in the same manner; but all experience goes the other way.

A good deal has been made of the question of facility of access, in regard to which we would remark that, after all, Manchester is not such a very enormous place; it is also a very network of tram-lines, and seeing the distance which patients already travel to reach its Infirmary, we think that the perfection of the hospital as a curative agent is far more important than an absolutely central position. As for the doctors, they will go to the hospital wherever it is placed. It is over four miles from Harley Street to the London Hospital, and we never heard that the London ever suffered from want of men. What the Manchester people should do is to choose the healthiest site, and on it build the healthiest hospital. If a place like Halifax, to mention a town in their own neighbourhood, can afford to purchase thirteen acres of land for a hospital of three hundred beds, and to build single story pavilions, Manchester will but cover itself with ridicule if, in deference to some ill-defined sentiment as to the old place, it should build a hospital on a site which has been condemned by experts in sanitation and in hospital construction. The cat is let out of the bag by Mr. Brockbank, who, writing to the *Manchester Guardian*, says, "Let us at the same time have an elevation for the new building worthy of the site." While we are anxious in regard to sanitation, people in Manchester seem to be troubling their souls about an elevation! At all hazards their Piccadilly must be preserved. If, then, the inevitable must be accepted, even though it savour of the absurd, and if the Manchester people still determine to stick to the old site for their new infirmary, then they must be prepared to pay the cost for their fancy. It must not be supposed for a moment that a hospital can be put up at the same expense on a foul site, in the midst of bad surroundings, as it could be on clean ground amid purer air. The sick must not be allowed to suffer, and, expensive though it may be, some attempt must be made to prevent the badness of the site telling, more than is inevitable, on the progress of the patients in the new Manchester Royal Infirmary. The whole of the soil on which the old infirmary has stood should be excavated, removed, and replaced by pure earth from a distance, before the new building is erected, and if, in consequence of the smallness of the area available, it should be necessary to pile up ward upon ward, and erect a tall edifice like the model dwellings with which London is being defaced, then care must be taken not only to ensure free currents of air beneath the pavilions, but so to break up the superimposed stories with alternating air spaces as to prevent, as far as possible, continuity of atmosphere throughout the building. All this we fear means ruinous expense; but people must pay for their fancy. Our point is that the sick should not suffer for it.

We still have hope, however, that the people of Manchester will accept the wiser course. At a meeting of the trustees of the Royal Infirmary, held March 26th, the proposal to rebuild on the present site was negatived, and the whole matter was referred to a joint committee; and if this committee will but shut its eyes to local prejudices, and consider solely the advantage of the sick, we have no doubt as to the result.

